# A convenient and sensitive allergy test: IgE crosslinking-induced luciferase expression in cultured mast cells

**DOI:** 10.1111/j.1398-9995.2010.02363.x

**Published:** 2010-10

**Authors:** R Nakamura, Y Uchida, M Higuchi, R Nakamura, I Tsuge, A Urisu, R Teshima

**Affiliations:** 1 Division of Novel Foods and Immunochemistry, National Institute of Health ScienceTokyo; 2 Department of Pediatrics, Fujita Health University School of Medicine, ToyoakeAichi, Japan

**Keywords:** allergy test, food allergy, IgE, luciferase, mast cell

## Abstract

**Background:**

For the detection of allergen-specific IgE in sera, solid-phase IgE-binding assays like the CAP test are commonly used. Although such immunochemical methods are very sensitive, they frequently produce false positives. Degranulation of the human IgE receptor (FcεRI)-transfected rat mast cell (RBL) lines seems to be a possible indicator for human IgE, but spontaneous mediator release from these cells in the presence of human sera is not negligible.

**Methods:**

The nuclear factor of activated T-cells (NFAT)-responsive luciferase reporter gene was stably transfected into human FcεRI-expressing RBL-SX38 cells. One established clone (RS-ATL8) was sensitized with 1 : 100 dilution of sera from patients with egg white allergy and then stimulated with purified or a crude extract of egg white allergen.

**Results:**

Sensitization with 15 pg/ml IgE was sufficient to detect IgE crosslinking–induced luciferase expression (EXiLE) by anti-IgE stimulation. Allergen-specific EXiLE was elicited by as little as 1 fg/ml of egg white protein without cytotoxicity. There was a good correlation between results with EXiLE and oral food challenge tests on patients with egg allergy (*P* = 0.001687, Fisher's exact test). The measured values of EXiLE and the CAP test also correlated well (*R* = 0.9127, Spearman's test).

**Conclusion:**

The EXiLE test using RS-ATL8 cells is a promising *in vitro* IgE test to evaluate the biological activity of the binding between IgE and allergens.

Food allergy occurs in 5–10% of infants and preschool children and 1–2% of school children in Japan (1). Ideally, oral food challenge (OFC) tests would be available for each patient with food allergy to determine his/her responsive allergens. However, it is often the case that OFC tests are avoided because of the patient's age, physical condition or clinical history (2). The *in vitro* allergen-specific IgE test using patients’ sera, like ImmunoCAP (CAP test), is widely used for the initial screening purposes for responsive allergens. The CAP test is a highly automated, convenient and very sensitive method (sub ng/ml) for detecting serum IgE binding to allergens (3). However, results of specific IgE binding to allergens cannot always be translated into a clear diagnosis, especially in the cases of food allergy (4, 5). Such clinically irrelevant results in serum IgE tests can be partly explained by cross-reactive carbohydrate determinants (CCDs) (5). The CCD-specific IgE in patients’ sera can bind to the carbohydrate residue(s) in the allergen. However, if the carbohydrate determinant has only one site per allergen, such binding between the IgE and allergen would not induce mast cell activation because of failure to crosslink the high-affinity IgE receptor (FcεRI) on the mast cells (6).

High-affinity IgE receptor is a heterotetrameric receptor composed of an α subunit, a β subunit, and a homodimer of γ subunit (7). Among these subunits, only the α subunit has a binding ability to IgE, and expression of only the α subunit is sufficient for high-affinity binding to human IgE (8). So far, there are no useful human mast cell lines that express abundant FcεRI and grow well (9–12). Therefore, human FcεRI-overexpressing rodent mast cell lines may be a useful system for reflecting crosslinking of FcεRI on mast cells triggered by patients’ IgE and specific allergens. We and several other groups have transfected a rat basophilic leukemia-derived mast cell line, RBL-2H3, with the α subunit gene or a complete set of α/β/γ subunit genes of the human FcεRI, and analyzed the usefulness of the system (13–17). Among these cell lines, α/β/γ-transfected RBL cells were found to have the potential to be sensitized with diluted patients’ sera and degranulate after the addition of specific allergens. In particular, RBL-SX38 cells, generated by Wiegand et al. (14), were found to be the most effective (18). However, human serum was cytotoxic at high concentrations (typically, more than 1 : 10–1 : 20). To avoid cytotoxicity, investigators had to sufficiently dilute serum (16), or remove the cytotoxic factors by adsorbing the sera to wild-type RBL-2H3 cells (15, 17, 18). These treatments could reduce the IgE concentration in diluted sera, or increase experimental uncertainty through increased manipulations. Moreover, the level of degranulation was relatively low after such treatments, so artificial ‘accelerators’ of degranulation, such as an adenosine analogue (15) or deuterium oxide (D_2_O; 12–14), were required to measure meaningful responses. These compounds have been reported to potentiate the degranulation of mast cells (19–22), but the addition of high concentrations of D_2_O increased spontaneous mediator release from these cells (18, 20, 21).

Crosslinking of FcεRI on mast cells will also induce marked gene expression of chemokines, cytokines, and other proteins (23). A number of transcription factors participate in such responses, and we previously demonstrated that nuclear factor of activated T-cells (NFAT) appeared to play one of the most important roles in FcεRI crosslinking–induced gene expression in RBL-2H3 cells (24).

Here, we show that the introduction of a NFAT-responsive luciferase reporter gene into human FcεRI-expressing RBL cells is a convenient method for detecting IgE crosslinking–induced mast cell activation with low-background and high sensitivity. We designated the novel method as the ‘EXiLE’ test; IgE crosslinking–induced luciferase expression test.

## Materials and methods

### Cells

RBL-SX38 cells, expressing the human FcεRI α/β/γ-subunits, were a kind gift from Dr Kinet at Beth Israel Deaconess Medical Center (Boston, MA), and were maintained as previously reported (14). The NFAT-regulated luciferase reporter gene plasmid containing hygromycin resistance gene were purchased from Biomyx (San Diego, CA, USA). The plasmid was linearized by *Bgl* I digestion, and was transferred into RBL-SX38 cells using Lipofectamine 2000 (Invitrogen, Rockville, MD, USA) following the manufacturer's protocol. Stable transfectants were selected using 600 μg/ml hygromycin. The RS-ATL8 cell line, the highest luciferase responder subclone after stimulation with 10 nM phorbol myristate acetate and 10 μM ionomycin, was established by limiting dilution and was grown in minimum essential medium (MEM) (Invitrogen) supplemented with 10% heat-inactivated fetal calf serum (FCS; Nichirei Biosciences, Tokyo, Japan), penicillin/streptomycin, GlutaMAX-I, 1.2 mg/ml geneticin, and 200 μg/ml hygromycin (Invitrogen).

### Human sera and allergens

Patients with suspected egg allergy referred to our hospital for investigation were enrolled in the study. Most of the patients had atopic dermatitis and asthma. Informed consent was obtained from patients, their parents, or both to collect and investigate serum samples further. The study was approved by the institutional review board of National Institute of Health Sciences and Ethics Committee of Fujita Health University School of Medicine. All sera were collected before food challenge to measure total and allergen-specific IgE levels using ImmunoCAP. Every patient was subjected to double-blind, placebo-controlled food challenges with heated egg and was judged positive if he or she was unable to eat the equivalent of a whole boiled egg, as described previously (25). Pooled sera from healthy donors were purchased from Cosmo Bio (Tokyo, Japan). All sera were stored at −80°C until use. Purified ovalbumin (OVA; grade V, >98%) was from Sigma (St. Louis, MO, USA). To determine the allergen detection limit, a 5 mg/ml egg white-extracted protein (EWP) solution (Greer labs, Lenoir, NC, USA) was used. Egg white extract (100 mg/ml of freeze-dried egg white powder in stock solution) prepared for the scratch diagnostic tests was purchased from Torii Pharmaceutical (Tokyo, Japan) for the experiments to determine the correlations between the allergy tests.

### Luciferase assay

RS-ATL8 cells (5 × 10^4^ cells/50 μl/well) were plated onto a clear-bottom white 96-well plate (ViewPlate Perkin Elmer, Waltham, MA, USA), and were incubated for several hours at 37°C in a 5% CO_2_ incubator. Then, 5 μl of partly diluted (11 : 100) or serially diluted sera in MEM containing 10% FCS was added to the supernatant (final dilution in the former case, 1 : 100). After overnight incubation, the cells were washed once with sterile PBS and then stimulated for 3 h at 37°C in a 5% CO_2_ incubator with allergens diluted in MEM containing 10% FCS (50 μl/well). After stimulation, 50 μl of luciferase substrate solution containing cell lysis reagent (ONE-Glo, Promega Corp., Tokyo, Japan) was added to the cells, and chemiluminescence was measured using an EnVision multilabel plate reader (Perkin Elmer). Luciferase expression levels are represented as the fold increase of light units compared with the background expression, after subtraction of a blank control (without cells).

### β-Hexosaminidase assay

Stimulation-induced β-hexosaminidase release from RBL cells was measured as described previously with some modifications (13). RBL-SX38 cells were plated and sensitized with serially diluted sera as described earlier. After overnight incubation, the cells were washed twice with normal PIPES buffer (140 mM NaCl, 5 mM KCl, 0.6 mM MgCl_2_, 1.0 mM CaCl_2_, 5.5 mM glucose, 0.1% BSA and 10 mM PIPES, pH 7.4). OVA (1 μg/ml) or goat anti-human IgE antibody (Bethyl laboratories, Montgomery, TX, USA) diluted in prewarmed PIPES buffer was added to the cells (50 μl/well). After incubation for 30 min at 37°C, supernatants were collected, and residual cells were lysed with 0.2% Triton X-100. Activity of β-Hexosaminidase in the medium and within the cells was determined by a fluorometric assay using 4-methylumbelliferyl-N-acetyl-β-D-glucosaminide as a substrate (0.1 mM in 100 mM citrate, pH4.5). The reaction was stopped with 0.25 M glycine buffer after 30 min incubation at 37°C. The plate was read on a Fluoroskan Ascent plate reader (Thermo Fisher Scientific, Waltham, MA, USA) using 380 nm excitation and 440 nm emission filters. Degranulation levels are represented as follows; 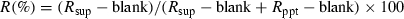 where *R* is the degranulation level; *R*_sup_ is released enzyme activity in the supernatant; *R*_ppt_ is residual enzyme activity within the cell; blank is the buffer control.

### LDH release assay

Cytotoxicity of human sera on RS-ATL8 cells was measured by a lactate dehydrogenase (LDH) release assay using the Cytotoxicity Detection Kit^PLUS^ (Roche Diagnostics K.K., Japan) following the manufacturer's protocol, and an EL340 (BioTek Instruments, Winooski, VT, USA).

### Statistics

The cytotoxic effect of human serum was analyzed using a 1-way anova followed by Dunnett's test compared with negative controls without human serum. Fisher's exact test was performed to analyze correlations between the OFC and EXiLE or CAP tests. Spearman's rank correlation test was performed to compare the EXiLE and CAP test.

## Results

### RS-ATL8 cells

**Figure 1:**
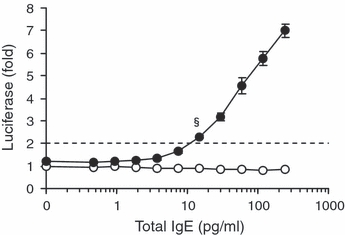
Detection limit of IgE crosslinking–induced luciferase expression. RS-ATL8 cells were sensitized with serial dilutions of healthy donor's serum in 10% Fetal calf serum-containing medium. The total IgE concentration in the original serum was determined by ELISA. After overnight incubation, the cells were stimulated with (closed circles) or without (open circles) 1 μg/ml of anti-human IgE for 3 h. Dashed line, two-fold level of the background (control without serum) luciferase expression. §IgE = 15 pg/ml (0.006 IU/ml). Data are means ± SEM (*n* = 4).

In the present study, we transfected an NFAT-responsive luciferase reporter gene into human FcεRI α/β/γ-expressing RBL-SX38 cells. We obtained 16 clones after hygromycin resistance selection, and found that clone number 8 was the highest responder to phorbol ester and ionomycin (data not shown). This clone was designated as RS-ATL8; RBL-SX38 cell stably transfected with NFAT-Luciferase clone 8. The cells were sensitized with serially diluted healthy donor's serum overnight, and then stimulated with 1 μg/ml goat anti-human IgE antibody for 3 h. A dose-dependent increase in EXiLE in the RS-ATL8 cells was observed (Fig. 1). The original serum contained 99 ng/ml (41 IU/ml) of IgE as revealed by ELISA. If an expression level of two-fold background (without serum) was set as a cut-off, EXiLE by anti-IgE crosslinking exceeded the cut-off at 15 pg/ml of human IgE (0.006 IU/ml). In our preliminary results, sensitization and stimulation periods of 16 h (overnight) and 3 h, respectively, resulted in the highest luciferase expression (data not shown).

### Comparison of EXiLE to the β-hexosaminidase release method

**Figure 2:**
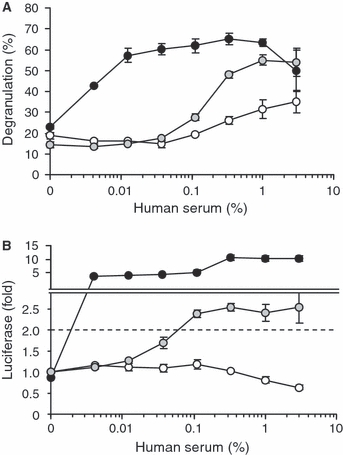
Comparison of degranulation and luciferase expression. RBL-SX38 cells (A) and RS-ATL8 cells (B) were sensitized with serial dilutions of egg white allergy patient's serum (total IgE, 12 700 IU/ml; egg white specific IgE, >100 U_A_/ml) overnight. Cells were stimulated with 1 μg/ml ovalbumin (shaded circles), 1 μg/ml anti-human IgE (closed circles), or solvent alone (open circles). Solvent does not include any degranulation accelerator such as D_2_O. Degranulation after 30-min stimulation (A), and luciferase expression after 3-h stimulation (B) are shown. Dashed line in B, two-fold level of background (control without serum) luciferase expression. Data are means ± SEM (*n*= 4 in a, *n* = 3 in b).

As previously reported, RBL-SX38 cells are capable of effectively degranulating after sensitization using sera from patients with peanut allergy and the addition of peanut allergen (15, 19). However, human serum (1 : 10) was cytotoxic to the cells, so it had to be cross-adsorbed against wild type RBL-2H3 cells prior to the sensitization of RBL-SX38 cells (15, 19). Moreover, when degranulation assays were performed, it was usual for an ‘accelerator’ of degranulation, like D_2_O or 5′-N-ethylcarboxamide, to be added to the medium (15–18). Here, RBL-SX38 cells or RS-ATL8 cells were similarly sensitized with serially diluted serum from egg allergy patient's (total IgE, 12 700 IU/ml; egg white specific IgE, >100 U_A_/ml), and stimulated with 1 μg/ml of OVA and anti-human IgE for 30 min in PIPES buffer or for 3 h in 10% FCS-containing MEM, respectively (Fig. 2). RBL-SX38 cells degranulated following stimulation with a specific allergen even without a degranulation accelerator (Fig. 2A). However, as serum concentration increased, spontaneous release increased, particularly in cases of more than 0.1% serum. The minimum and maximum spontaneous release was 14.6% and 34.9% of total enzyme, respectively. Meanwhile, the background luciferase expression levels were gradually decreased at >0.3% serum (Fig. 2B). EXiLE by OVA exceeded 2.0 at 0.1% serum, and was >2.0 until 3% serum. Anti-IgE stimulation elicited dramatic increases in luciferase in RS-ATL8 cells throughout the serum concentration range tested (0.004–3%), whereas β-hexosaminidase release by anti-IgE stimulation decreased nearly to the spontaneous release levels at higher serum concentrations (Fig. 2A).

### Appropriate serum dilution for EXiLE in RS-ATL8 cells

**Figure 3:**
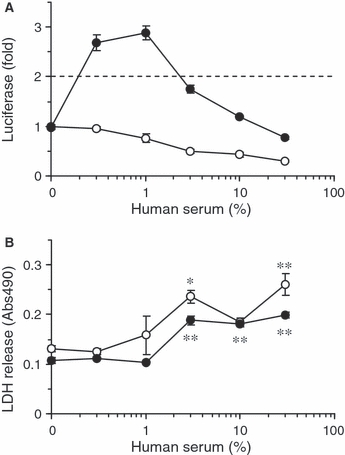
Appropriate serum dilution. RS-ATL8 cells were sensitized with serial dilution of pooled healthy donor's serum overnight. Cells were stimulated with 1 μg/ml anti-human IgE (closed circles), or solvent alone (open circles) for 3 h. (A) IgE crosslinking–induced luciferase expression by anti-human IgE; (B) Cytotoxicity of human serum on the RS-ATL8 cells revealed by lactate dehydrogenase release. Dashed line in a, two-fold level of background (control without serum) luciferase expression. Data are means ± SEM (*n* = 4). *P < 0.05, **P < 0.01 (Dunnett's test).

To determine the appropriate dilution factor for human sera, anti-human IgE-induced luciferase expression and cell viability after sensitization of RS-ATL8 cells with serially diluted healthy donor's serum were measured. LDH is a stable cytosolic enzyme and is released to the medium if the plasma membrane is damaged (26). Anti-IgE stimulation elicited a marked increase in the luciferase expression in RS-ATL8 cells sensitized with 0.3% and 1% serum; however, at higher serum concentrations (≥3–30%), the expression decreased depending on the serum concentration (Fig. 3A). The decrease was also seen in the nonstimulated controls. Such decreases in luciferase expression inversely correlated to LDH release (Fig. 3B), suggesting that human serum has a cytotoxic activity on the RS-ATL8 cells. In preliminary experiments, this effect could be decreased by more than 50% by heating the sera to 56°C for 30 min, suggesting heat-labile factors (maybe complement) cause the cytotoxicity. However, heat-inactivation was not to be performed in our experiments because IgE is also heat-labile (27). Therefore, we concluded that a 1 : 100-dilution was the most appropriate to determine EXiLE without cytotoxicity.

### Detection limit of allergen by means of EXiLE

**Figure 4:**
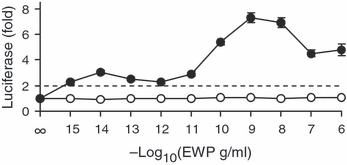
Detection limit of egg white allergen. RS-ATL8 cells were sensitized with 1 : 100-diluted healthy donor's serum (open circles) or egg allergy patient serum (closed circles) overnight. Cells were stimulated with the indicated concentrations of egg white proteins (EWP) diluted in 10% Fetal calf serum-containing medium for 3 h. Dashed line, two-fold level of background (0 g/ml EWP) luciferase expression. Data are means ± SEM (*n* = 4).

We next tried to determine the detection limit of egg white allergen using the patient's serum. RS-ATL8 cells were sensitized with 1 : 100-diluted patient serum or healthy donor serum overnight, and were stimulated with EWP serially diluted in MEM containing 10% FCS for 3 h. Figure 4 shows that EXiLE by EWP peaked at 1 ng/ml and exceeded 2.0 at 1 fg/ml. The lot-to-lot reproducibility and effect of cell passage number on the EXiLE test were determined; 1 ng/ml EWP induced a 7.3 ± 1.1 (mean ± SEM) fold increase in luciferase expression across six different time points (Fig. S1). It was impossible to perform the same experiment on β-hexosaminidase release from RBL-SX38 cells, because significant spontaneous release (49%) was induced by sensitization with the 1 : 100-diluted serum (data not shown).

### Comparison of EXiLE to OFC and CAP tests

Nineteen sera from suspected egg-allergy patients who had been diagnosed by the OFC test (double-blind placebo-controlled food challenge test) with heated egg and also by the egg white–specific serum IgE test (CAP test) were subjected to the EXiLE test for comparison. RS-ATL8 cells were sensitized with 1 : 100-diluted patients’ sera, and stimulated with 0, 1, 10, 100, and 1000 ng/ml of EWP, and EXiLE by EWP was calculated by assuming the background expression with no allergen was 1.0, and was judged to be positive if the maximum EXiLE value (Max EXiLE) was ≥2.0. Table 1 summarizes the results; 12 of 19 patients were positive in the OFC test, 18 of 19 were positive in the CAP test (class 2 as a cut-off; specific IgE ≥ 0.70 U_A_/ml, a level indicated in the manufacturer's instruction), and 12 of 19 were positive in the EXiLE test. Among 12 OFC positive patients’ sera, 11 and 12 were predicted to be positive by EXiLE and CAP tests, respectively, while among 7 OFC negative patients’ sera, 6 and 1 were predicted to be negative by EXiLE and CAP tests, respectively. As revealed by Fisher's exact test, the OFC and EXiLE tests correlated well (*P* = 0.001687), whereas the OFC and CAP tests did not (*P* = 0.3684).

**Table 1:** EXiLE in RS-ATL8 cells sensitized with egg-allergy patients' sera

							EXiLE (fold) by EWP (ng/ml)							
											
Patient	Age, Sex	OFC* test	totIgE (IU/ml)	CAP (U_A_/ml)	CAP class	CAP* test†	1	10	100	1000	Max EXiLE (fold)	EXiLE score‡	EXiLE* test§	Anti-IgE EXiLE (fold)
#79	6, M	**+**	1922	**76.8**	5	**+**	**2.3**	**3.4**	**4.5**	**3.5**	**4.5**	4	+	16.7
#77	13, M	**+**	1273	**68.6**	5	**+**	**4.1**	**8.8**	**9.5**	**7.5**	**9.5**	4	+	17.9
#78	9,F	**+**	844	**48.3**	4	**+**	**4.8**	**8.3**	**8.4**	**8.6**	**8.6**	4	+	17.6
#80	6,M	**+**	381	**35.1**	4	**+**	1.5	**3.0**	**3.9**	**5.0**	**5.0**	3	+	12.0
#74	9,M	**+**	2663	**29.9**	4	**+**	**3.9**	**3.3**	**4.5**	**4.8**	**4.8**	4	+	20.9
#90	14, F	**+**	12323	**25.6**	4	**+**	1.5	**2.5**	**2.8**	**2.8**	**2.8**	3	+	16.7
#84	5,M	**+**	331	**13.2**	3	**+**	1.5	1.7	**2.3**	1.9	**2.3**	2	+	7.84
#83	10, M	**+**	772	**8.98**	3	**+**	1.3	1.7	**2.5**	**3.2**	**3.2**	2	+	17.7
#85	8,M	**+**	619	**5.57**	3	**+**	1.6	**2.5**	**3.4**	**3.1**	**3.4**	3	+	24.2
#81	10, F	**+**	795	**4.54**	3	**+**	1.5	**2.2**	1.8	**2.7**	**2.7**	3	+	19.5
#76	6,M	**+**	509	**3.91**	3	**+**	1.4	1.7	**2.0**	**2.2**	**2.2**	2	+	23.0
#94	9,F	**+**	653	**2.99**	2	**+**	0.8	1.3	1.3	1.5	1.5	0	−	11.8
#73	9,F	−	7339	**33.4**	4	**+**	1.8	**2.2**	**2.2**	1.9	**2.2**	3	+	26.9
#67	13, M	−	22600	**4.46**	3	**+**	1.0	1.2	1.4	1.6	1.6	0	−	14.2
#82	10, M	−	1470	**3.45**	2	**+**	1.2	1.2	1.5	1.4	1.5	0	−	16.7
#95	5,F	−	578	**2.03**	2	**+**	1.1	1.2	1.3	1.5	1.5	0	−	10.0
#75	6,M	−	1006	**1.17**	2	**+**	0.8	0.9	0.9	1.0	1.0	0	−	25.0
#100	9,M	−	5356	**0.89**	2	**+**	0.9	1.3	1.5	1.4	1.5	0	−	23.0
#99	13, M	−	475	<0.34	0	−	1.1	1.1	1.3	1.5	1.5	0	−	7.13

RS-ATL8 cells were sensitized with l : 100-diluted egg-allergy patients' sera overnight, and stimulated with the indicated concentrations of EWP or 1 μg/ml anti-IgE for 3 h.

* Correlation between OFC and CAP tests was *P* = 0.3684, and that between OFC and EXiLE tests was *P* = 0.001687 (Fisher's exact test).

† CAP test was considered positive if the class was ≥2 (i.e. ≥0.70 U_A_/ml)

‡ EXiLE score varies from 0 to 4. Score 1, 2, 3, and 4 means its EXiLE exceeds 2.0 at EWP concentrations of 1000, 100, 10, and 1 ng/ml, respectively. Score 0 means negative.

§ EXiLE test was judged to be positive if Max EXiLE was more than the cut-off level (2.0).

Values greater than cut-off levels are represented in Bold. EWP, Egg white proteins; EXiLE, IgE crosslinking–induced luciferase expression; OFC, oral food challenge.

**Figure 5:**
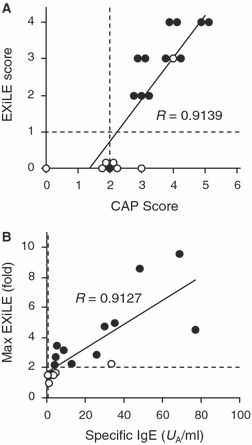
Correlation between CAP test and IgE crosslinking–induced luciferase expression (EXiLE) test. Results in Table 1 are depicted in graphic form. Correlation between CAP classes and EXiLE scores (A), and that of allergen-specific IgE concentration and Max EXiLE (B) are shown. Closed and open circles, subjects from oral food challenge positive and negative, respectively. Vertical and horizontal dashed lines, cut-off levels of the CAP and EXiLE test, respectively. *R*-values of Spearman's rank correlation test were 0.9139 (*P* < 0.001) in A, and 0.9127 (*P* < 0.001) in B.

The EXiLE scores 1, 2, 3, and 4 correspond to the minimum concentrations of responsive EWP; 1000, 100, 10, and 1 ng/ml, respectively, while 0 means a nonresponder. We analyzed the correlation between the values and scores of the CAP and EXiLE tests by a Spearman's rank test. Figure 5 illustrates that there are very good correlations between the CAP and EXiLE scores (*R* = 0.9319, *P* < 0.001), and between the CAP (specific IgE concentration) and Max EXiLE values (*R* = 0.9127, *P* < 0.001).

Max EXiLE values and the ratio of specific/total IgE concentrations were also compared and found to correlate well, but the Spearman's correlation coefficient was slightly lower (*R* = 0.8561, *P* < 0.001) than that between CAP and Max EXiLE. These results seemed to be consistent with a previous study by Dibbern Jr. et al. (15), looking at the degranulation of RBL-SX38 cells.

## Discussion

In the present study, we have established a stable luciferase-reporting humanized RBL clone, RS-ATL8, derived from the previously described RBL-SX38 cells (14, 15). RBL-SX38 cells released β-hexosaminidase on activation following sensitization with serum from patients with egg allergy and stimulation with EWP, without any degranulation accelerators (Fig. 2A). However, there was significant spontaneous release from serum-sensitized but nonstimulated cells, which varied from patient to patient (data not shown). In a previous report, Ladics et al. described that humanized RBL cells, including RBL-SX38 cells, were not conducive to using a broad range of allergic subjects sera, because of difficulties in generating consistent, robust responses (15). In our system, human sera can be diluted 1 : 100, avoiding serum cytotoxicity (Fig. 3B), and generate consistent responses without any artificial accelerators with very high sensitivity (≥15 pg/ml IgE). In appropriate conditions, our system can detect at least 1 fg/ml of allergen (Fig. 4). It is noteworthy that the medium used here is supplemented with 10% FCS, which includes incredible amounts of nonspecific proteins, ensuring the robustness of the system. Indeed, antigen-specific luciferase expression was observed either by purified allergen (Fig. 2B) or by a mixture of egg white allergens (Fig. 4) with the same patient's serum. So far, nine food allergens (egg white, egg yolk, milk, wheat, buckwheat, peanut, shrimp, crab, and sesame) and three inhaled allergens (mite, cedar pollen, and cat dander) were capable of inducing EXiLE in RS-ATL8 cells sensitized with specific patients’ sera. Some of the EXiLE experiments undertaken for the screening of allergens are shown in Fig. S2. As allergens are in the liquid phase in our system, the degree-of-freedom for testing allergens appears to be higher than in a solid phase system like CAP.

As shown in Table 1, comparative analysis of the EXiLE test to the OFC test demonstrated that the predicted results with the EXiLE test correlated well to the final diagnosis obtained with the OFC test (*P* = 0.001687, Fisher's exact test), whereas the results with the CAP test had no significant correlation to the OFC test (*P* = 0.3684). The low correlation between the CAP and OFC tests here could be partially explained by the high sensitivity of the CAP test. In CAP tests, a class of 2 (≥0.70 U_A_/ml) is usually adopted for a cut-off point following the manufacturer's instructions. However, Ando et al. (25) recently reported that the optimal cut-off point of egg white–specific IgE was 2.82 and 7.38 U_A_/ml, for the OFC tests with raw and heated egg white, respectively. In addition, Komata et al. (28) also reported that the 95% probability was achieved at 30.0 U_A_/ml for patients with egg white allergy who were aged ≥2. Considering these aspects, the class of 2 might be too strict to exclude false positives, at least for the patients in the present study. If a CAP class of 3 (≥3.50 U_A_/ml) was set as a cut-off in Table 1, the correlation of the results with the CAP test and OFC test would increase to a significant level (*P* = 0.009546). Therefore, the CAP test is so sensitive that it seems to be suitable for the first screening of allergen-specific IgE in the sera.

Both of the scores (Fig. 5A) and values (Fig. 5B) from the EXiLE and CAP tests correlated well. These results strongly suggest that an increase in the luciferase expression observed in RS-ATL8 cells reflects crosslinking of the antigen-specific IgE bound to FcεRI on the mast cells. Therefore, these three systems seemed to correlate well, but there were several specific differences in the results between them (Table 1). Compared with the OFC results, the EXiLE test predicted one false-negative (#94) and one false-positive (#73). Reasons for this are unclear, but the false-negative result might be because of the higher detection limit of the EXiLE system, as serum #94 contained no more than 2.99 U_A_/ml specific IgE. Another possibility is that other substances, like chemokines and cytokines, can induce histamine release from human basophils (29, 30). As RS-ATL8 cells only have human FcεRI, the cells would not be activated through IgE-independent mechanisms like these. This might also be an explanation for the false-positive case. If neutralizing IgG antibodies (31) against EWP were present *in vivo*, it would not be strange that oral challenge did not show marked symptoms in the patient, whereas antibodies other than IgE in the patient's serum will be washed away from the medium before the addition of specific antigen to RS-ATL8 cells in the present study. A qualitative change in epitopes by heat and/or digestion could be another possibility.

One noteworthy example is patient #67, whose serum contained 4.46 U_A_/ml (class 3) of egg white–specific IgE, but can eat eggs with no allergic symptoms. With this serum, EXiLE levels were gradually increased to 1.6 depending on EWP concentration, but did not exceed the 2.0 cut-off level (Table 1). Considering the substantially high level of total IgE in his serum (22 600 IU/ml), most of the specific IgE seems to be biologically meaningless cross-reactive antibodies, lacking the ability to activate mast cells and basophils (5, 32). It is important to distinguish such patients from the CAP-positive patient group because food avoidance is unnecessary and would lower the quality of life for such patients (33). The EXiLE test could be suitable for this purpose, screening allergen-specific IgE in sera following immunochemical *in vitro* tests like CAP.

This novel *in vitro* technique might be applicable to: (i) second screening of allergen-specific IgE in the serum following CAP tests, (ii) cross-reactivity tests between known allergens and potential allergens (safety assessment of gene-modified crops), (iii) standardization of allergen extracts for clinical usage, (iv) screening of novel allergens and/or epitopes, (v) detection of small amounts of allergens in foods, (vi) epidemiological study of allergens using a serum bank, and (vii) high-throughput screening for the seed compounds of anti-allergic drugs.

## Abbreviations

EWP: Egg white proteins
EXiLE: IgE crosslinking–induced luciferase expression
FCS: Fetal calf serum
FcεRI: High-affinity IgE receptor
LDH: Lactate dehydrogenase
NFAT: Nuclear factor of activated T-cell
OFC: oral food challenge
OVA: Ovalbumin
RS-ATL8: RBL-SX38 cell stably transfected with NFAT-luciferase, clone 8

## Supporting Information

**Figure S1.** Lot-to-lot reproducibility and the effect of passage number on the EXiLE test.

**Figure S2.** Allergen screening using EXiLE test.
